# An Unusual Complication of Measles Infection in a Pediatric Patient: A Case Report of Measles-Induced Hemophagocytic Lymphohistiocytosis (HLH) From Jordan

**DOI:** 10.7759/cureus.48144

**Published:** 2023-11-02

**Authors:** Dana Kanaan, Mohammad N Al-Khazali, Israa W Khalid, Faisal Abu-Ekteish, Suleimman Al-Sweedan

**Affiliations:** 1 Department of Pediatrics, Jordan University of Science and Technology, Irbid, JOR; 2 Department of Pediatrics, Princess Rahma Teaching Hospital, Jordanian Ministry of Health, Irbid, JOR

**Keywords:** viruses, pediatrics, vaccination, hemophagocytic lymphohistiocytosis (hlh), measles

## Abstract

Measles is a highly contagious infection that leads to many serious complications. Despite the significant global effort to eradicate it, it still represents a major threat due to suboptimal vaccination coverage, especially after the coronavirus disease 2019 (COVID-19) pandemic that affected all routine childhood vaccinations. One of its fatal complications, which has been reported a few times in the literature, is hemophagocytic lymphohistiocytosis (HLH). We discuss a case of a 14-month-old unvaccinated female patient who developed measles-induced HLH and was treated with intravenous immunoglobulins (IVIG) and steroids; unfortunately, she developed multiorgan failure and passed away before chemotherapy could be initiated.

## Introduction

Measles is a vaccine-preventable, potentially fatal infection [1]; it had been responsible for more than two million deaths every year worldwide before the introduction of the measles vaccine. Despite the fact that the measles vaccine is highly effective, outbreaks continue to occur due to suboptimal vaccination coverage in many parts of the world [2,3]. The incidence of measles in Jordan has varied widely over the years, but it tended to rise from 2002 to 2022 and peaked in 2022; this rise in cases was associated with a decline in vaccination coverage [4]. Measles is known to cause serious early and late complications, such as pneumonia, encephalitis, and subacute sclerosing panencephalitis (SSPE)[1]. Other rare complications have also been described in the literature, such as pulmonary embolism, pancreatitis, and hemophagocytic lymphohistiocytosis (HLH) [5-7].

HLH is a rare syndrome, characterized by a highly stimulated but ineﬀective immune response. The mechanism involves an inherited or acquired defect in the handling of infectious, cancerous, or autoimmune stimuli that causes serious systemic inflammatory response [8]. Viral-induced HLH is a well-described entity in literature, but measles-induced HLH has been sparsely reported [7,9-13] and hence needs further studies and attention, especially in the setting of recurrent measles outbreaks worldwide. We describe a case of a 14-month-old unvaccinated child who developed HLH after a measles infection.

## Case presentation

The patient was a 14-month-old unvaccinated female, born to healthy non-consanguineous parents, who was not known to have any medical illness and was deemed satisfactory in terms of developmental milestones. Her past medical history was significant for two admissions due to diarrheal illness at the age of five months and 12 months, both resolved without sequelae. She was first seen at a local hospital with a fever and diarrhea of three days' duration. Her physical examination was normal except for moderate dehydration, and her laboratory findings were only significant for mild leukopenia. She was admitted for hydration, but her family discharged her against medical advice within a few hours of admission.

Two days later, she was brought again to the emergency department due to a persistent high-grade fever that had not resolved since discharge, and associated with a new-onset cough and skin rash. On physical examination, her vital signs were as follows - temperature: 39 °C, O_2_ saturation: 95% on room air, heart rate: 140 beats/minute, respiratory rate: 40 breaths/minute, blood pressure: 90/59 mmHg, and her Glasgow Coma Scale (GCS) score was 15/15. She had a toxic appearance, with a generalized erythematous maculopapular rash. The chest exam revealed diffuse crackles bilaterally. Her abdominal exam showed abdominal distention with hepatosplenomegaly. No conjunctivitis or lymphadenopathy was noted. Her initial laboratory workup showed pancytopenia, elevated liver enzymes, and low ESR. A chest X-ray was done at admission and revealed a right upper lobe infiltration. CT scan of the abdomen was also done and showed hepatosplenomegaly, moderate abdominal and pelvic fluid accumulation, and minor bilateral pleural effusion.

She was admitted to the PICU for cardiopulmonary support and started empirically on IV meropenem, vancomycin, and acyclovir for presumed sepsis and doxycycline for possible rickettsia. She was also started on 2 g/kg IV immunoglobulins (IVIG) to be given over two days for possible post-COVID multisystemic inflammatory syndrome (MIS-C). Over the next two days, her clinical status deteriorated as she started requiring oxygen support, and she developed a generalized tonic-clonic seizure; a CT brain was done and no hemorrhage on the brain edema was seen. Her follow-up laboratory workup showed no improvement. This raised suspicion of HLH, and hence she was transferred to our hospital, which is a tertiary hospital with an oncology unit, for further management and workup.

Upon arrival at our hospital, she was found to be critically ill, in shock with unstable vitals, a temperature of 40 °C, a respiratory rate of 50 breaths/minute, a heart rate of 130 beats/minute, and a blood pressure of 85/45 mmHg. Her Oxygen saturation was 85% on room air and her GCS score was 12/15. An inspection of the skin also showed a generalized erythematous maculopapular rash. Koplik spots were also seen on the buccal mucosa. The chest exam revealed diffuse crackles bilaterally with normal heart sounds.

The laboratory investigation performed on admission showed pancytopenia, elevated liver enzymes, elevated INR, hypofibrinogenemia, high LDH, and elevated serum ferritin. The lipid profile (mainly triglycerides) was highly elevated as well. Peripheral blood smear, kidney function test, and electrolytes were within the normal range. Initial blood culture did not yield any pathogens and results of the infectious workup including cerebral spinal fluid analysis and culture and urine culture were negative as well. Bone marrow aspiration and biopsy could not be performed as the patient was clinically unstable.

Measles IgM antibodies had been detected serologically (measles IgM of 40; reference range for antibodies: positive above 11). Viral serology was performed for EBV, CMV, parvovirus B19, COVID-19 by PCR, and rickettsia, and all results were negative. Table 1 summarizes the laboratory findings during the course of admission. As the patient fulfilled the HLH criteria (Table 2), the HLH-2004 protocol was followed, and dexamethasone was administered in addition to wide-spectrum antibiotics. The patient also received multiple blood component transfusions, including factor 7, cryoprecipitate, packed RBC, platelets, and fresh frozen plasma.

**Table 1:** Laboratory findings during admission WBC: white blood cells; Hb: hemoglobin; Plt: platelets; PT: prothrombin time; aPPT: activated partial thromboplastin time; ALT: alanine transaminase; AST: aspartate aminotransferase; LDH: lactate dehydrogenase; ESR: erythrocyte sedimentation rate; BUN: blood urea nitrogen

Variables	Day 1	Day 3	Day 5	Day 6	Normal range
WBC (10^3^/mm^3^)	3.7	1.97	2.25	2.08	4–11
Neutrophils %	20%	13.2%	9%	31%	60–75%
Lymphocytes %	70%	70%	76%	47%	20–45%
Hb (g/dl)	12.2	11.2	10.5	7.3	10.9–15.0
Plt (10^3^/mm^3^)	194	50	36	48	150-400
PT (seconds)	-	38	60	55	14.5–16.5
aPTT (seconds)	-	40	46	39	25–45
INR	-	-	-	5	0.8–1.1
Ferritin (ng/mL)	-	-	3965	-	7–142
Fibrinogen (mg/dl)	-	-	151	135	200–400
AST (U/L)	-	2702		5212	0–32
ALT (U/L)	-	5753		3241	0–33
Albumin (g/dl)	-	2.9	2.7	2.5	3.5–5.0
LDH (U/L)	-	-	1552	>750	143–370
Triglyceride (mmol/L)	-	-	2.48	3.26	1.7–2.8
ESR (mm/hr)	-	4	4	-	0–15
Sodium (mmol/L)	137	136	128	135	135–145
Creatinine (umol/L)	13	12	18	18	27–88
BUN (mmol/l)	1.7	3.7	3.7	6	1.45–6.78

**Table 2:** HLH-2004 diagnostic criteria and patient findings WBC: white blood cells; Hb: hemoglobin; HLH: hemophagocytic lymphohistiocytosis; NK: natural killer cells; SIL-2R: soluble interleukin-2 receptor; BM: bone marrow; N/A: not available

	Patient findings	HLH-2004 diagnostic criteria (at least five of eight main criteria should be fulfilled)
Fever	Reached 40 °C, for 7 days	>38.5 °C for more than 7 days
Splenomegaly	Present	Radiological or physical exam
Cytopenia		2 or 3 hematopoietic lineages
WBC	2.08 x 10^3^/mm^3^	
Hb	7.3 g/dl	<9 g/dl
Platelets	48 x 10^3^/mm^3^	<100 x 10^3^/mm^3^
Neutrophils	868/µL	<1000/µL
Triglyceride	3.26 mmol/L	>3 mmol/L
Fibrinogen	135 mg/dl	<150 mg/dl
Ferritin	3965 ng/mL	>500 ng/ml
SIL-2R	N/A	>2400 U/ml
Hemophagocytosis	N/A	Present in BM, spleen, or lymph node
NK cell activity	N/A	Low or absent

Ten hours post-admission, the patient’s respiratory functions and level of consciousness deteriorated dramatically. Unfortunately, the patient did not survive this critical condition and passed away 24 hours after admission with pulmonary hemorrhage and cardiopulmonary arrest documented as the cause of death.

## Discussion

Fever, hyperferritinemia, and pancytopenia associated with multiorgan failure in a patient should raise strong concerns about HLH [8]. Early diagnosis and treatment are crucial for reducing mortality in these patients; however, due to the rarity of the disease and the lack of specificity in the clinical signs, its diagnosis is often challenging.

Numerous illnesses have been linked to HLH, and both familial and sporadic cases are frequently triggered by infections, which are usually viral. As a result, it is difficult to distinguish between acquired and familial HLH when identifying an infection [14]. While secondary HLH is the most probable diagnosis in our patient given a negative family history of primary HLH, parental consanguinity, and family history of unexplained pediatric death, we cannot definitely be sure without a genetic study [14].

Treatment for an infectious agent(s)-triggered HLH should target the following aspects: elimination of current triggers, regulation of the hyper-inflammatory response, and supportive treatment for the consequences and organ failures that develop over the clinical course [15]. As viral infections are the leading cause of secondary HLH, the management of common viruses that are linked to HLH like EBV has been discussed thoroughly in the literature [14,15]. Measles-induced HLH has been reported in six studies in the literature (Table 3). All of them involved patients below the age of 19 years; all of them presented with pneumonia, and five of them were immunocompetent patients [7,9,11-13]. Various treatment approaches were reported to be effective in managing those cases, Komatsuda et al. have reported a patient who achieved remission with pulse steroid therapy alone [7], and Lagousi et al. have described a four-month-old female patient whose condition improved with steroid and IVIG alone [9]; also, Joshi et al. have reported a school-aged female who survived with only supportive treatment [12]. Our patient's condition did not improve with immunoglobulins and steroids alone, which were started on day three and day five of illness, respectively. Unfortunately, she deteriorated before she was started on chemotherapy. A timely diagnosis and the start of appropriate medication could increase the survival among HLH patients, especially those with other unmodifiable poor prognostic factors [high ferritin (>2,000 µg⁄L), respiratory failure, coagulopathy, neutropenia, and hypoalbuminemia], which were all present in our patient [16].

**Table 3:** Cases of measles-induced hemophagocytic lymphohistiocytosis (HLH) reported in the literature

Study	Patient age	Gender	Comorbidities	Treatment	Clinical outcome
Huang et al. [10]	17 years	Male	High-risk acute lymphoblastic leukemia in remission	IV methylprednisolone, IV immunoglobulin, etoposide, plasma exchange	Died
Lagousi et al. [9]	4 months	Male	None	IV dexamethasone, IV immunoglobulin	Survived
Komatsuda et al. [7]	18 years	Male	None	IV methylprednisolone	Survived
Yamamoto et al. [11]	8 years	Male	None	IV methylprednisolone, cyclosporin A	Survived
Joshi et al. [12]	Below 13 years	Female	None	Antibiotics only	Survived
Pearl et al. [13]	2 years	Male	None	VP-16, epipodophyllotoxin	Died

A major risk factor that our patient had is that she was unvaccinated. Although mandatory measles vaccination was introduced in Jordan in 1981 [17], measles cases in Jordan have fluctuated over the years. Two peaks were recorded between 2000 and 2022 [4]: one in 2013, which was attributed to the Syrian refugee crisis [18], and another in 2022 with 457 cases, which represents the highest number of cases ever recorded in Jordan since 2000 [4]. This rise in cases was associated with a decline in vaccination coverage; the official estimate of coverage in 2022 as reported by WHO was 86% for the first dose and 80% for the second dose, compared to 87% and 99%, respectively, in 2019 [4]. This decline in vaccination coverage was mostly related to the effect of the 2020 COVID-19 pandemic as it placed a heavy burden on healthcare systems in Jordan as well as in many other countries, leading to a disruption in childhood vaccination services [19,20].

## Conclusions

Given the increasing number of measles cases in Jordan and worldwide, better knowledge and awareness of probable measles-induced HLH, its early detection, and the early start of appropriate therapy are essential to save patient lives. More effort is required to raise vaccination rates, manage outbreaks, and enhance herd immunity. This strategy is essential for protecting vulnerable people, mainly young infants who have not yet received their vaccination, as well as immunocompromised patients who are most at risk of developing measles-related HLH.
